# Diagnostic accuracy of capnovolumetry for the identification of airway obstruction – results of a diagnostic study in ambulatory care

**DOI:** 10.1186/s12931-019-1067-1

**Published:** 2019-05-15

**Authors:** Christina Kellerer, Neele Jankrift, Rudolf A. Jörres, Klaus Klütsch, Stefan Wagenpfeil, Klaus Linde, Antonius Schneider

**Affiliations:** 1 TUM School of Medicine, Institute of General Practice and Health Services Research, Technical University of Munich, Orleansstraße 47, 81667 Munich, Germany; 2 Institute and Outpatient Clinic for Occupational, Social and Environmental Medicine, Ludwig-Maximilians-Universität München, Munich, Germany; 3 Institute for Medical Biometry, Epidemiology and Medical Informatics (IMBEI), Saarland University, Homburg, Germany

**Keywords:** Capnovolumetry, Airway obstruction, Asthma, COPD, Diagnostic study, Sensitivity, Specificity, ROC analysis, Area under the curve (AUC)

## Abstract

**Background:**

One of the known weaknesses of spirometry is its dependence on patients’ cooperation, which can only partially be alleviated by educational efforts. Therefore, procedures less dependent on cooperation might be of value in clinical practice. We investigated the diagnostic accuracy of ultrasound-based capnovolumetry for the identification of airway obstruction.

**Methods:**

Consecutive patients from a pulmonary outpatient clinic were included in the diagnostic study. As reference standard, the presence of airway obstruction was evaluated via spirometry and bodyplethysmography. Capnovolumetry was performed as index test with an ultrasound spirometer providing a surrogate measure of exhaled carbon dioxide. Receiver operating characteristic (ROC) analysis was performed using the ratio of slopes of expiratory phases 3 and 2 (s3/s2) ≥ 0.10 as primary capnovolumetric parameter for the recognition of airway obstruction. Logistic regression was performed as secondary analysis to identify further useful capnovolumetric parameters. The diagnostic potential of capnovolumetry to identify more severe degrees of airway obstruction was evaluated additionally.

**Results:**

Of 1400 patients recruited, 1287 patients were included into the analysis. Airway obstruction was present in 29% of patients. The area under the ROC-curve (AUC) of s3/s2 was 0.678 (95% CI 0.645, 0.710); sensitivity of s3/s2 ≥ 0.10 was 47.7 (95% CI 42.7, 52.8)%, specificity 79.0 (95% CI 76.3, 81.6)%. When combining this parameter with three other parameters derived from regression analysis (ratio area/volume phase 3, slope phase 3, volume phase 2), an AUC of 0.772 (95% CI 0.743, 0.801) was obtained. For severe airway obstruction (FEV_1_ ≤ 50% predicted) sensitivity of s3/s2 ≥ 0.10 was 75.9 (95% CI 67.1, 83.0)%, specificity 75.8 (95% CI 73.3, 78.1)%; for very severe airway obstruction (FEV_1_ ≤ 30% predicted) sensitivity was 86.7 (95% CI 70.3, 94.7)%, specificity 72.8 (95% CI 70.3, 75.2)%. Sensitivities increased and specificities decreased considerably when the combined capnovolumetric score was used as index test.

**Conclusions:**

Capnovolumetry by way of an ultrasound spirometer had a statistically significant albeit moderate potential for the recognition of airway obstruction in a heterogeneous population of patients typically found in clinical practice. Diagnostic accuracy of the capnovolumetric device increased with the severity of airway obstruction.

**Trial registration:**

The study is registered under DRKS00013935 at German Clinical Trials Register (DRKS).

## Background

Asthma [1] and chronic obstructive pulmonary disease (COPD) [2] are frequent respiratory diseases associated with airway obstruction. Commonly, the diagnosis of these disorders is determined on the basis of clinical history and spirometry. Detailed guidelines and instructions for spirometry are widely available [3] and programs for improving the quality of assessments, particularly by the training of nurses, have been implemented. Despite this, the validity of spirometric results is not rarely insufficient in clinical practice [4, 5], most often due to difficulties of the patients to follow the instructions of forced maneuvers. Clinical experience shows that there are always patients not capable of performing correct breathing maneuvers even after repeated instruction. Therefore, methods requiring a low degree of cooperation could be helpful for establishing a diagnosis in these patients.

There are several methods with low demands regarding cooperation, such as the interrupter technique [6], impulse oscillometry (IOS) [7], and capnometry based on the measurement of the concentration of exhaled carbon dioxide (CO_2_) [8]. Capnometry has been studied for about 70 years [9] but is still not integrated into clinical routine. Part of this might be due to the fact that additional equipment in form of CO_2_ sensors was required. Meanwhile, however, techniques have been developed to estimate CO_2_ from ultrasound signals used for spirometry solely by software algorithms, without the need for a CO_2_ sensor [10]. This offers the possibility to perform capnographic measurements via suitable spirometers during a phase of quiet breathing prior to spirometry.

**Fig. 1:**
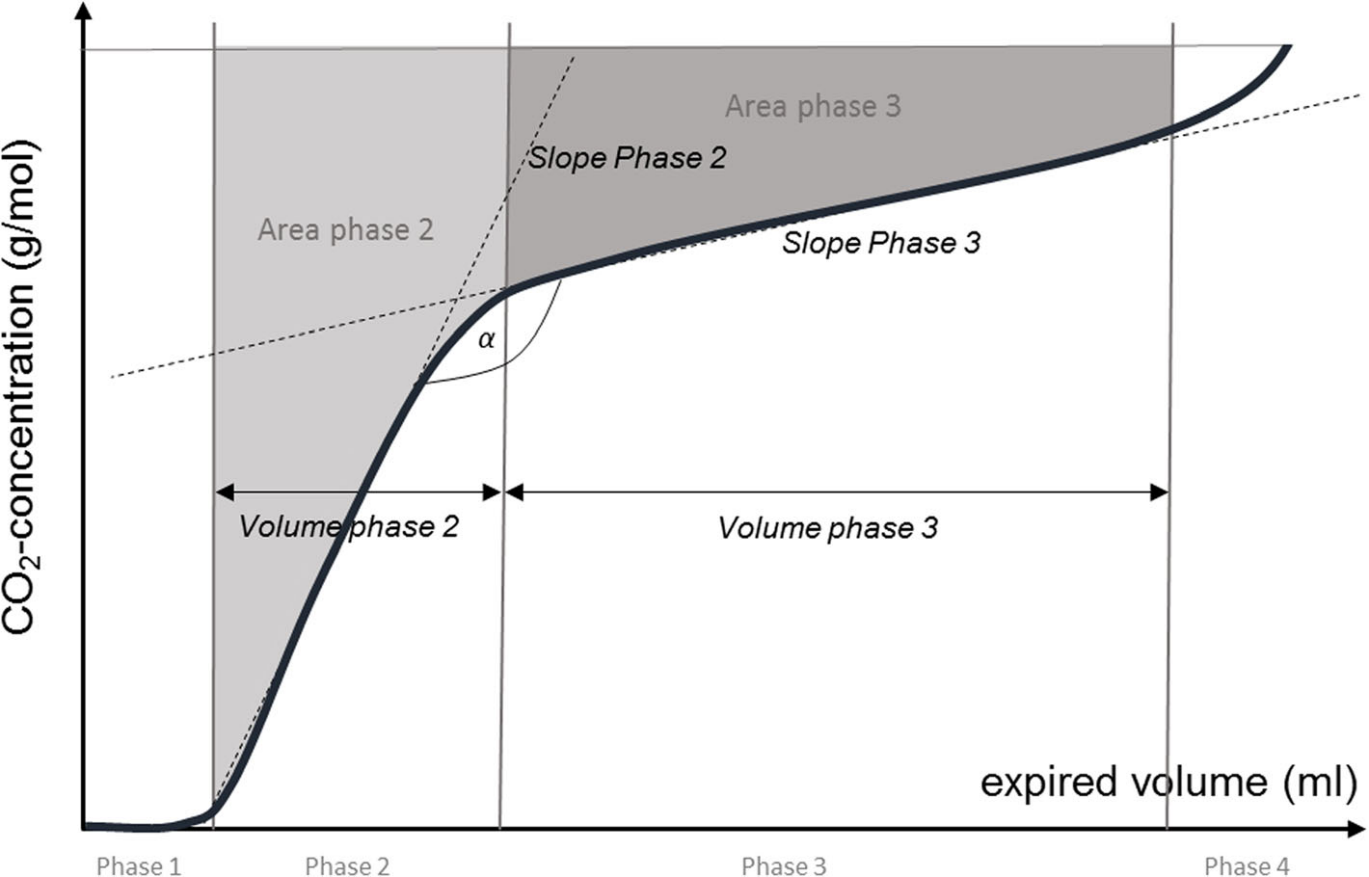
Tracing of a volume-based capnogram during a single expiration. The capnogram is divided into four phases: phase 1, the CO_2_-devoid volume of the dead space; phase 2, transition between airway and alveolar gas; phase 3, alveolar part; phase 4, final emptying of the lung (in tidal breathing normally absent or inconspicuous). (A) Parameters of capnographic measurement: s2 and s3 represent the slopes (concentration vs. volume) of the expiratory CO_2_-curve in phase 2 and phase 3, respectively. The volume expired during phase 2 or phase 3 is termed ‘volume phase 2’ or ‘volume phase 3’. In order to compute the parameters ‘area phase 2’ and ‘area phase 3’ a horizontal line at the end-tidal CO_2_ concentration is drawn. The area above the CO_2_ concentration curve bounded by the horizontal line in phase 2 and phase 3, respectively, represent the parameters ‘area phase 2’ and ‘area phase 3’. These areas are complements of the areas under the curve. The angle alpha (α) is formed by the slopes of phases 2 and 3

Capnographic measurements can be described by various parameters, and a number of investigations have addressed the question, which of these parameters are suited to assess the presence of airway obstruction (e.g. [8, 11, 12]). One study [10] suggested a high diagnostic accuracy especially for the ratio of slopes of phases 3 and 2 that are obtained when the expiratory CO_2_ concentration is plotted against volume (capnovolumetry, see Fig. 1), thereby recommending this parameter for further evaluation. The clinical setting most promising for capnovolumetry might be primary care, in which the most basic diagnostic question refers to the presence of airway obstruction. Most of the available studies, however, investigated small, highly selected samples of patients which could lead to biased estimation [13]. Using the ratio of slopes in capnovolumetry as primary parameter, we therefore aimed to quantify the diagnostic accuracy of capnovolumetry for the recognition of airway obstruction in a large sample of unselected patients under ambulatory care conditions. For this purpose, we studied patients who visited a pulmonary outpatient clinic and were well characterized regarding their clinical and functional status.

## Methods

### Study design and sample

This prospective diagnostic study was performed between February and April 2018 in a pulmonary outpatient clinic led by six pneumologists in Augsburg (Germany). In Germany, outpatient clinics are organized as private practices of specialists in primary care, which can be visited by patients for diagnostic investigation and treatment without referral. We included 1400 consecutive patients attending the clinic for their first diagnostic work-up or follow-up evaluations and giving oral and written informed consent. Exclusion criteria were age less than 18 years and/or inability to understand the German language, without further requirements. Patients’ diagnoses were based on the evaluation of all functional and clinical information available, including chest x-rays, bronchial provocation challenges and bronchodilator tests, as documented in the patients’ files. All diagnoses including those of comorbidities were taken from these files. The specialists were blinded to the results of the capnographic measurements.

Based on previous studies, we expected a prevalence of airway obstruction of 20% [14]. In a pilot study using the ratio of slopes of phases 3 and 2 (s3/s2), the sensitivity was 90% and the specificity 86% for the detection of airway obstruction at the cut-off s3/s2 ≥ 0.10 [15]. A power calculation based on these assumptions revealed that at least 1280 patients were needed to establish a sensitivity and specificity of 80% each with a 95% confidence interval of ±5% [16]. We expected incomplete data in about 10% of patients and therefore aimed to include 1400 patients. The study was approved by the Ethical Committee of the Technical University of Munich (TUM). The study protocol was registered in the German Clinical Trials Register (DRKS00013935).

### Test methods

#### Capnovolumetric index test

Capnovolumetric measurements were based on ultrasound estimation of the expiratory carbon dioxide (CO_2_) concentration using the device SpiroScout (Software LFX 1.8.0, Ganshorn, Niederlauer, Germany). The estimation was performed via molar mass measurement, as the ultrasound technology not only allows to detect relative changes in sound velocity that are proportional to flow rate but also the determination of absolute sound velocity which depends on air density and therefore CO_2_ concentration. Patients performed quiet tidal breathing over at least 10 breathing cycles while sitting and wearing a noseclip. The verbal instruction given to them was only to avoid deep breaths or panting.

The device measures airflow velocity via the delay and acceleration of ultrasound signals and at the same time the absolute velocity of signals which is related to the composition of air. Taking into account temperature and humidity by adequate models, the concentration of CO_2_ can be derived from the measured molar mass of the exhaled air. Compared to conventional CO_2_ measurement no additional hardware is needed, since the assessment is achieved by software from available signals.

The parameters describing the shape of the expiratory CO_2_ curve against expired volume are explained in Fig. 1. Each parameter provided by the device represents a mean value of all recorded breathing cycles. The indices most important for the present study were the slopes of expiratory phases 2 (s2) and 3 (s3), as well as their ratio s3/s2. In addition, the expired volumes of the two phases and corresponding areas under the concentration-volume curve could be defined.

#### Reference standard

Spirometric and bodyplethysmographic measurements were routinely performed within the assessment of patients, following established criteria for spirometry [17] and bodyplethysmography [18, 19]. Whether airway obstruction was present, was decided on the basis of both spirometric and bodyplethysmographic results. Obstruction was assumed if the z-score of the ratio (FEV_1_/FVC) of forced expiratory volume in 1 second (FEV_1_) and forced vital capacity (FVC) was less than − 1.645 [20] or, in case of a normal ratio, either specific airway resistance (sRaw) or airway resistance (Raw) were above 1.2 kPa*s or 0.3 kPa*s/l, respectively [18]. Each patient with airway obstruction was reviewed by an expert team (RAJ, AS) to cross-check the diagnostic decision making.

### Data analysis

Baseline data is presented descriptively. Regarding lung function and capnovolumetric parameters, differences between the groups of patients with and without airway obstruction were assessed via the Mann-Whitney U test. Categorical variables were compared using the Chi-square statistics. For the evaluation of capnovolumetry as index test, the ratio (s3/s2) of slopes of phases 3 and 2 of the expiratory CO_2_ concentration curve versus volume was chosen. Using this parameter, receiver operating characteristic (ROC) curves for the recognition of airway obstruction were constructed and quantified via the area under the curve (AUC), its standard error of mean (SEM) and the corresponding 95% confidence interval (95% CI). Two-by-two contingency tables of capnovolumetric values vs. bodyplethysmographic diagnosis of airway obstruction were prepared using different levels of s3/s2 as cut-off. Sensitivities and specificities were calculated for the previously identified cut-off of 0.10 [15] and for the Youden-Index (cut-off at the highest sum of sensitivity and specificity) [21]. 95% confidence intervals were calculated using Wilson’s method [22].

In secondary analyses we addressed two questions, firstly the diagnostic accuracy (AUC, sensitivity, specificity) for the detection of airway obstruction in groups of patients with different degree of obstruction as quantified by FEV_1_ being ≤80%, ≤50% or ≤ 30%, and secondly the role of capnovolumetric parameters in addition to the ratio of slopes of phases 3 and 2. The relative importance of capnographic parameters provided by the device was determined by stepwise multiple logistic regression analysis in the total population. To reduce problems arising from collinearity, the maximum number of parameters kept for prediction was limited to four. For the purpose of this analysis, the slope s3/s2 was log-transformed after addition of 0.05; this was done to account for zero values and to achieve a distribution as close to normal as possible. Similarly, the slope s3 was log-transformed after addition of 0.03. The score provided by the logistic regression analysis was then used for ROC analysis in analogy to s3/s2, and this was done in the total population as well as the groups of patients with different degrees of airway obstruction according to FEV_1_.

Capnovolumetric parameters are known to be affected by anthropometric charcteristics and breathing pattern, especially tidal volume [23]. We thus addressed their dependence on tidal volume, age, height and gender in a sensitivity analysis, using standard multiple linear regression methods. Using the predicted values from these analyses, we then checked whether normalization of capnographic parameters improved the results regarding the recognition of airway obstruction.

All analyses were performed using the software package SPSS (Version 25, IBM, Armonk, NY, USA), and the level of statistical significance was assumed at *p* = 0.05.

## Results

### Study population

**Fig. 2:**
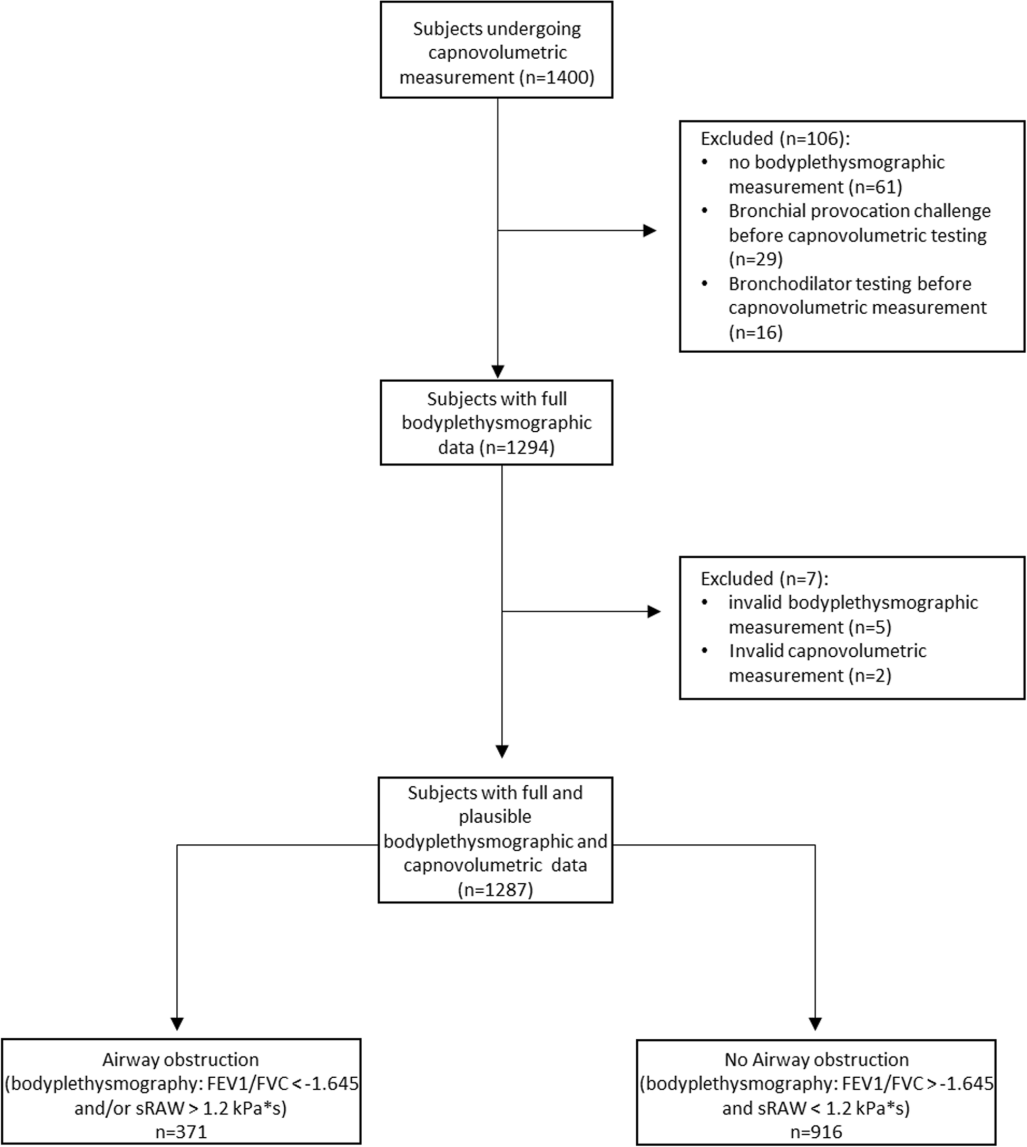
Flow-chart of the selection process leading to the final population of 1287 patients. A total of consecutive 1400 patients underwent capnovolumetry. Patients who turned out to have had bronchial provocation challenges or bronchodilator testing prior to capnography due to organizational reasons were excluded from analysis (*n* = 45). Moreover, patients who did not undergo bodyplethysmographic and spirometric measurements (*n* = 61) were excluded. Five patients were excluded due to low quality of their bodyplethysmographic data, and two patients based on invalid capnovolumetric measurements

A total of 1287 patients could be included into the analysis, of whom 371 (29%) showed signs of airway obstruction according to spirometry and/or bodyplethysmography (Fig. 2). The characteristics of the participants are shown in Table 1, demonstrating that the group of patients with airway obstruction was significantly different from the group without obstruction in all measures except body mass index (BMI). The parameters assessed in capnovolumetry are explained in Fig. 1. Regarding the major capnovolumetric parameters (Table 2), the primary target parameter s3/s2 showed significantly higher values in patients with airway obstruction compared to those without. Similarly, all other parameters determined by capnovolumetry, except the expired volume of phase 2, showed significant differences between patients with and without airway obstruction.

**Table 1:** Baseline characteristics

Parameter		Presence of airway obstruction		
	All (*n* = 1287)	no (*n* = 916)	yes (*n* = 371)	Comparison between groups (*p*-value)
Gender (m/f)	589/698	385/531	204/167	< 0.001
BMI (kg/m^2^)	26.9 (23.7; 31.1)	27.0 (23.9; 31.2)	26.7 (23.4; 35.3)	0.288
Age (y)	59.0 (47.0; 70.0)	56.0 (42.8; 77.0)	62.0 (53.0; 79.0)	< 0.001
FEV_1_ z-Score	−0.92 (−1.98; − 0.05)	−0.47 (− 1.09; 0.24)	− 2.47 (− 3.16; − 1.65)	< 0.001
FEV_1_/FVC	74.9 (66.2; 81.4)	78.6 (74.1; 83.8)	59.8 (48.9; 70.9)	< 0.001
FEV_1_/FVC z-Score	− 0.63 (− 1.72; 0.25)	− 0.12 (− 0.74; 0.54)	−2.44 (− 3.3; − 1.77)	< 0.001
FVC z-Score	−0.58 (− 1.41; 0.20)	−0.37 (− 1.05; 0.38)	− 1.22 (− 2.02; − 0.33)	< 0.001
sRaw (kPa*s)	0.54 (0.27; 1.04)	0.39 (0.20; 0.62)	1.51 (0.85; 2.54)	< 0.001
Raw (kPa*s/l)	0.20 (0.10; 0.35)	0.14 (0.08; 0.23)	0.45 (0.28; 0.71)	< 0.001
FRC_pleth_ z-Score	−0.40 (− 1.16; 0.57)	− 0.66 (− 1.32; 0.08)	0.30 (− 0.56; 1.32)	< 0.001
Smoking status (current/ex/never)	253/485/536	154/303/447	99/182/89	< 0.001

The table shows absolute numbers in case of frequencies, median values and quartiles in case of continuous parameters. The groups were compared with each other using the Mann-Whitney-U-test, the categorical variables were compared using the Chi-square statistics. FEV_1_, forced expiratory volume in one second; FVC, forced vital capacity; sRaw, specific airway resistance (effective); FRC_pleth_, functional residual capacity determined by bodyplethysmography. Z-Scores were computed using the respective prediction equations [20]. The groups were statistically significantly different from each other in all parameters except BMI. Among the 371 patients with airway obstruction, 108 (29%) had asthma, 223 (60%) COPD, 24 (7%) the diagnosis of other respiratory diseases (such as restrictive disorders, pneumonia or other infections, pleural diseases, lung tumor, bronchiectasis), while in 16 (4%) of these patients no respiratory disease was found. Among the 916 patients without airway obstruction, 325 (35%) had asthma verified by bronchial provocation, 243 (27%) suffered from other respiratory diseases (such as restrictive disorders, pneumonia or other infections, pleural diseases, lung tumor, bronchiectasis, chronic bronchitis), and 348 (38%) had no respiratory disease

**Table 2:** Parameters of capnographic measurements

Parameter		Presence of airway obstruction		
	All (*n* = 1287)	No (*n* = 916)	yes (*n* = 371)	Comparison between groups (*p*-value)
Slope phase 2, s2 (g/mol*l)	2.89 (2.12; 3.81)	3.04 (2.23; 3.92)	2.59 (1.85; 3.43)	< 0.001
Slope phase 3, s3 (g/mol*l)	0.17 (0.10; 0.30)	0.16 (0.09; 0.27))	0.21 (0.12; 0.34)	0.001
log(s3)	−0.72 (−0.92; − 0.49)	−0.74 (− 0.96; − 0.54)	−0.64 (− 0.85; − 0.44)	< 0.001
Ratio s3/s2	0.06 (0.04; 0.10)	0.05 (0.03; 0.09)	0.09 (0.05; 0.14)	< 0.001
log(s3/s2)	−0.96 (−1.05; − 0.82)	− 1.00 (− 1.10; − 0.85)	− 0.85 (− 1.00; − 0.72)	< 0.001
alpha between s2 and s3 (°)	122.0 (116.0; 130.0)	120.5 (115.0; 127.0)	128.0 (119.0; 135.0)	< 0.001
Volume phase 2 (ml)	108.0 (91.0; 128.0)	109.0 (91.0; 129.0)	106.0 (89.0; 127.0)	0.134
Volume phase 3 (ml)	537.0 (392.0; 783.0)	522.5 (373.0; 751.8)	599.0 (432.0; 858.0)	0.002
Area/volume phase 2 (g/mol)	0.28 (0.24; 0.32)	0.27 (0.23; 0.31)	0.29 (0.25; 0.34)	< 0.001
Area/volume phase 3 (g/mol)	0.06 (0.05; 0.08)	0.05 (0.04; 0.07)	0.08 (0.06; 0.10)	< 0.001

The table shows median values and quartiles. The groups were compared with each other using the Mann-Whitney-U-test. For the explanation of parameters see Fig. 1. log(s3) is the logarithm (base 10) of the parameter s3, log(s3/s2) the logarithm (base 10) of the ratio s3/s2. Before taking the logarithm, the values of 0.03 and 0.05, respectively, were added to the parameter values in order to account for zero values and achieve a distribution being as close to normal as possible. The groups were statistically significantly different from each other in all parameters except volume phase 2

### ROC analysis

**Fig. 3:**
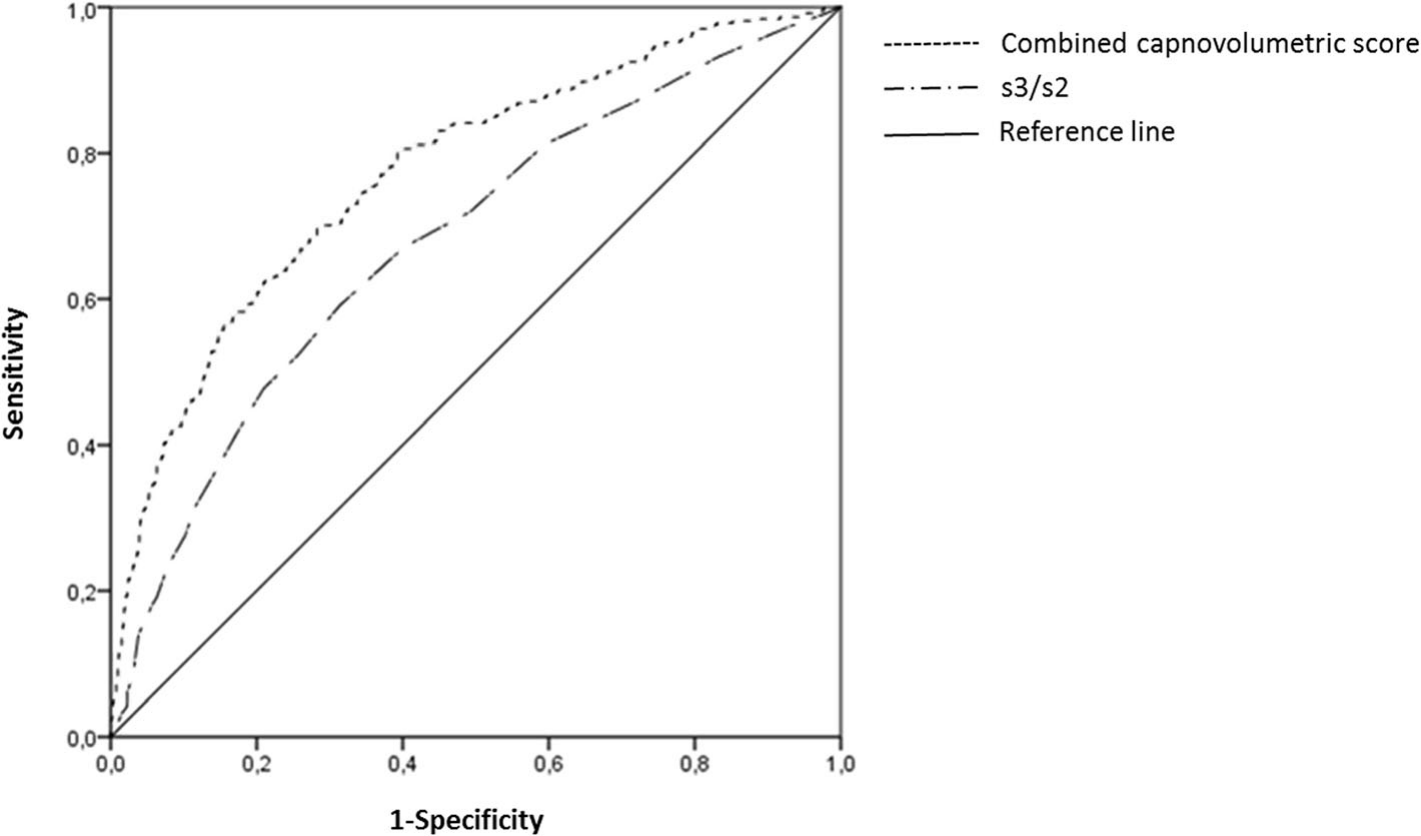
ROC-curves for the recognition of airway obstruction. The AUC for the ratio of slopes s3 and s2 (s3/s2) was 0.678 (95% CI 0.645, 0.710). The AUC for the combined capnovolumetric score derived from the area-to-volume ratio of phase 3, the logarithm of the slopes of phase 3, the volume of phase 2, and the logarithm of the ratio of slopes of phases 3 and 2 was 0.772 (95% CI 0.743, 0.801)

According to our primary study question, ROC analysis indicated a statistically significant ability of s3/s2 to detect the presence of airway obstruction (Fig. 3), as reflected in an AUC ± SEM of 0.678 ± 0.017 (95% CI 0.645, 0.710). The maximum Youden index was 0.277 and achieved for a cut-off value of 0.08. At this cut-off, sensitivity was 59.0% (95% CI 54.0, 63.9) and specificity 68.7% (95% CI 65.6, 71.6). When using the pre-defined cut-off value of 0.10 [15], sensitivity was 47.7% (95% CI 42.7, 52.8) and specificity 79.0% (95% CI 76.3, 81.6). More severe degrees of airway obstruction could be detected by s3/s2 at the pre-defined cut-off value of 0.10 with a higher diagnostic accuracy (Table 3).

**Table 3:** ROC Analyses of s3/s2 and the combined capnovolumetric score for different stages of airway obstruction

Airway obstruction (AO)	s3/s2				Combined capnovolumetric score			
	cut-off	sensitivity (%)	specificity (%)	AUC ± SEM (95% CI)	cut-off	sensitivity (%)	specificity (%)	AUC ± SEM (95% CI)
AO in spirometry / bodyplethysmography*	0.10	47.7 (42.7;52.8)	79.0 (76.3; 81.6)	0.678 ± 0.017 (0.645; 0.710)	0.26	69.8 (65.1; 74.7)	71.7 (67.7; 73.7)	0.772 ± 0.015 (0.743; 0.801)
FEV_1_ ≤ 80	0.10	46.2 (41.7; 50.8)	81.0 (78.2; 83.6)	0.699 ± 0.015 (0.669; 0.729)	0.26	64.8 (60.5; 69.3)	72.7 (69.0; 75.1)	0.743 ± 0.015 (0.713; 0.772)
FEV_1_ ≤ 50	0.10	75.9 (67.1; 83.0)	75.8 (73.3; 78.1)	0.851 ± 0.016 (0.820; 0.883)	0.26	88.9 (80.5; 92.8)	62.6 (60.6; 66.1)	0.854 ± 0.019 (0.818; 0.890)
FEV_1_ ≤ 30	0.10	86.7 (70.3; 94.7)	72.8 (70.3; 75.2)	0.887 ± 0.025 (0.838; 0.935)	0.26	93.3 (78.7; 98.2)	60.3 (57.6; 63.0)	0.860 ± 0.025 (0.810; 0.909)

The table shows the results of ROC analyses with s3/s2 and the combined capnovolumetric score for the recognition of different degrees of airway obstructions defined by restrictions in FEV_1_. FEV_1_, forced expiratory volume in one second; *AO* Airway obstruction, *AUC* area under the curve, *SEM* standard error mean, *95% CI* 95% confidence interval. (* z-Score FEV_1_/FVC < −1.645 and / or sRAW > 1.2 kPa*s)

### Identification of other relevant capnovolumetric parameters

Stepwise multiple logistic regression analysis performed in the total study population identified the four parameters ratio area/volume of phase 3, log(s3/s2), log(s3) and volume of phase 2 as dominant (*p* < 0.001 each) predictors, in that order (Table 4); thus the ratio s3/s2 chosen for the primary analysis was contained in this parameter set. The ROC-curve based on the derived probability scores is shown in Fig. 3, with an AUC ± SEM of 0.772 ± 0.015 (95% CI 0.743, 0.801). The Youden index was 0.415 using a cut-off value of 0.26 for the probability score, with a sensitivity of 69.8% (95% CI 65.1, 74.7) and a specificity of 71.7% (95% CI 67.7, 73.7). Compared to s3/s2, a significant improvement was achieved as indicated by the fact that the confidence intervals did not overlap. Additionally, the diagnostic accuracy of the combined capnovolumetric score at the determined cut-off value of 0.26 increased for the detection of severe degrees of airway obstruction (Table 3).

**Table 4:** Logistic regression analysis

Logistic regression analysis, dependent variable: airway obstruction (*n* = 1287)				
			95% Confidence interval	
Predictor	Regression coefficient	Standard error	Lower limit	Upper limit
Area/Volume phase 3 (g/mol)	31.805	3.290	25.3566	38.2534
log (s3/s2)	6.665	0.843	5.01272	8.31728
log(s3)	−4.092	0.542	−5.15432	−3.02968
Volume phase 2 (mL)	−0.019	0.003	−0.02488	−0.01312
Constant	2.328	0.795	0.7698	3.8862

The table shows the results of logistic regression analysis for the identification of relevant capnovolumetric parameters regarding the presence of airway obstruction. Only the four most relevant parameters were accepted; further parameters did not improve the result in a relevant manner. For the explanation of parameters see Fig. 1. The ratio of slopes of phases 3 and 2 (s3/s2) and the slope of phase 3 were logarithmically transformed prior to analysis in order to approximate normal distributions, and values of 0.03 and 0.05, respectively, were added before taking the logarithm in order to account for zero values. The predicted probability (P) of airway obstruction for each individual patient can be calculated as usual from the equation:

$$ P=\frac{{\mathrm{e}}^{\mathrm{L}}}{1+{\mathrm{e}}^{\mathrm{L}}} $$

in which L = constant + 31.805 * Area/Volume phase 3 + 6.665 * logs3s2 + (− 4.092) * logs3 + (− 0.019) * Volume phase 2. These predicted scores were used in the ROC analysis shown in Fig. 3

### Sensitivity analyses

In the capnovolumetric measurements, tidal volumes and breathing frequencies covered a broad range. According to multiple linear regression analyses, s3/s2 and the other three capnovolumetric parameters identified as most informative for the recognition of airway obstruction (see above) significantly depended on tidal volume, height, age and gender (*p* < 0.05 each). This raised the question whether the recognition of obstruction could be improved when expressing the measured values of the four parameters as percent of the values predicted from the regression analyses. However, no improvement could be achieved with this approach (AUC = 0.665 for s3/s2; AUC = 0.730 for the combination of the four capnovolumetric parameters).

## Discussion

Based on the fact that ultrasound-based capnovolumetry requires only minimal cooperation from the patient, it appears to be a promising candidate for the determination of airway obstruction in clinical practice. To our knowledge, this is the first study evaluating its diagnostic accuracy for this purpose in unselected patients under ambulatory care conditions. When using the pre-defined cut-off s3/s2 ≥ 0.10, sensitivity was 47.7% and specificity 79.0%, with an area under the ROC-curve of 0.678, indicating a considerably lower sensitivity in the present study compared to previous data [10, 15]. The result could be improved by using s3/s2 ≥ 0.08 as a slightly different cut-off value, but markedly and significantly only by the combination with three other indices describing the complex relationship between CO_2_ and volume. In patients with severe airway obstruction, its presence could be detected by s3/s2 and the combined capnovolumetric score with higher diagnostic accuracy. The s3/s2 ratio appears as an adequate measure at least of obstruction associated with ventilation inhomogeneity, as inhomogeneity should be associated with a steepening of the slope of phase 3 (unequal alveolar ventilation) and a flattening of the slope of phase 2 (mixing within bronchial compartment).

In the present study the sensitivity of s3/s2 regarding airway obstruction was considerably lower than reported previously [10, 15]. One reason might be found in differences between the study populations since the results by Ponto et al. [15] have been obtained in selected patients, which favours an overestimation of diagnostic accuracy [13]. The unselected population investigated in our study, although from a specialists’ practice, might be more representative of patients typically found in ambulatory care and thus provides a more realistic estimation of diagnostic accuracy. Differences in the study population appear to be particularly relevant when including patients with controlled asthma and a type of airway obstruction less associated with ventilation inhomogeneity than typical for COPD. A further reason for the decrease in sensitivity could have been that the commercially available device used by us utilized a modified computational algorithm compared to that used previously [15]. This might be relevant, as the derivation of a CO_2_ signal from the molar mass signal requires a number of non-trivial assumptions and computations, e.g. regarding the time course of temperature and humidity. Ultrasound spirometers have the benefit that no CO_2_ sensor is required, and thus no additional investment and risk of sensor instability over time. In case of a practical implementation it would be helpful to compare different ultrasound devices in diagnostic studies and to arrive at a consensus on optimal algorithms. Our study probably provides a realistic lower limit of the diagnostic accuracy that can be achieved under the conditions of clinical routine with current technology. This accuracy is encouraging at least for patients with more severe airway obstruction, given the problems arising from insufficient spirometric maneuvers.

Among the indices characterizing the CO_2_-volume curve, the ratio s3/s2 has the advantage to be easily interpretable in terms of ventilation inhomogeneity. If inhomogeneity is present, different regions of the lung show different CO_2_ concentrations, thus the concentration of exhaled CO_2_ in the alveolar phase 3 rises more steeply, i.e. the slope increases, when these regions are consecutively emptied. In parallel to the increase in the slope of phase 3, the slope of the bronchial phase 2 decreases, again as a result of inhomogeneous ventilation that smears out the concentration profile related to the airways; further details on capnovolumetry can be found in the literature [24]. Mismatches in the ratio of ventilation to perfusion may additionally contribute to changes in the slope particularly of phase 3. As a result, the ideal expiratory CO_2_ profile which is characterized by a very steep increase in the bronchial phase and a very flat curve in the alveolar phase, is distorted in two opposite ways, thereby explaining the superiority of the ratio s3/s2 compared to other parameters, as reported previously [10].

Accuracy could be considerably increased by using information beyond the ratio s3/s2, via inclusion of three further indices describing the shape of the expiratory CO_2_-curve (ratio area/volume of phase 3, slope of phase 3, volume of phase 2). This resulted in an area under the ROC-curve of 0.772, with sensitivity of 69.8% and specificity of 71.7%. The three additional parameters are also plausible from the pathophysiological point of view. As discussed above, the slope of phase 3 is closely related to the inhomogeneity of ventilation, which is linked to obstruction particularly in COPD [25, 26]. The same applied to the ratio of area to volume in phase 3, since, by definition, the area obtained by integrating the CO_2_ concentration over volume represents a total amount of exhaled CO_2_ due to inhomogeneity and thus the ratio to volume an average CO_2_ concentration change due to inhomogeneity. This was increased in obstructive patients, reflecting the concomitant deterioration of gas exchange and ventilation.

Of specific interest seemed the volume of phase 2 which was included in the logistic regression model, even though, in univariate analysis, it did not show a significant difference between the total groups of patients with and without airway obstruction. According to the algorithm by which this parameter is computed, its values should change in parallel to those of the classical Fowler dead space [27], provided that the threshold deadspace does not significantly change; this was the case in our study. Olsson et al. found that patients with mild airway obstruction not showing inhomogeneity of ventilation (e.g. with stable asthma) can exhibit a reduction of their bronchial space and thus of Fowler dead space [28]. To understand this issue we checked our data by stratification according to diagnoses, which indeed revealed the volume of phase 2 to be reduced in asthma, thereby contributing to the recognition of airway obstruction. In the present analysis, however, we focused on the basic clinical question of airway obstruction, without extension to differential diagnoses which would require information on clinical history and thus render a decision algorithm more complicated.

## Limitations and strength

The device used in the present study derived CO_2_ concentrations via the molar mass of exhaled air taking into account humidity and temperature. We cannot exclude slight deviations from the true CO_2_ concentration, as measurable, e.g., via a fast infrared sensor. At least in principle, a dependency of the temperature profile of exhaled air from airway obstruction [29] could affect the estimated values but this would not necessarily reduce their diagnostic usefulness. The parameter s3/s2 allows an intuitive interpretation reflecting the bend in the concentration-volume curve which separates phases 2 and 3. It was, however, inferior to the combined capnovolumetric score comprising the information from four parameters, whereby the critical cut-off point of the combined score was derived by secondary analysis. Therefore, this finding needs to be confirmed by a further diagnostic study. Such a study might also comprise different computational algorithms beyond the algorithm available in the commercial device in order to identify the most suited one.

A strength of the study was that we included a large group of patients consecutively within a large private practice of pneumologists. By reason of free access to health care, also regarding specialists, we think that this enabled us to determine the diagnostic accuracy with minor selection of patients under ´real world´ conditions. However, not all patients were included into the analysis, due to interventions performed prior to capnovolumetry. As this occurred in only few patients, it renders this selection secondary. As a major strength we consider the requirement that lung function was assessed via both spirometry and bodyplethysmography at about the same time as capnographic measurements.

## Conclusions

The results obtained in a large, unselected population from a pulmonary outpatient clinic indicate that capnovolumetry has a certain, but limited potential to indicate the presence of airway obstruction, at least if the previously recommended capnographic parameter s3/s2 and the currently available technology is used. However, by a combined score comprising four parameters diagnostic accuracy could be markedly improved. It is important to note that the diagnostic accuracy of the capnovolumetric device increased with the severity of airway obstruction. This diagnostic benefit should be considered in view of the low demands regarding the patients’ cooperation. There might be room for improvement by optimization of the computational algorithms, and the comparison of different devices/algorithms within further diagnostic studies would be necessary to enhance the efficiency of capnovolumetry.

## Abbreviations

95% CI: 95% confidence interval
AUC: Area under the curve
BMI: Body mass index
CO_2_: Carbon dioxide
COPD: Chronic obstructive pulmonary disease
FEV_1_: Forced expiratory volume in one second
FVC: Forced vital capacity
IOS: Impulse oscillometry
Raw: Airway resistance
ROC-curve: Receiver operating characteristic curve
s2: Slope of expiratory phase 2
s3: Slope of expiratory phase 3
s3/s2: Ratio of slopes of expiratory phases 3 and 2
SEM: Standard error of mean
sRaw: Specific airway resistance
